# Mechanistic role of CD82 palmitoylation in augmenting antitumor drug sensitivity via apoptosis regulation

**DOI:** 10.3389/fonc.2025.1699420

**Published:** 2025-12-03

**Authors:** Lanyan Li, Yaning Yu, Sirui Chen, Yihuan Zhang, Can Zhang, Xinyu Yao, Hongli Xu, Shihao Wu, Xue Gong, Weiping Han, Yu Zhang, Ying Li

**Affiliations:** Department of Clinical Laboratory, Second Affiliated Hospital of Dalian Medical University, Dalian, China

**Keywords:** CD82, palmitoylation, apoptosis, drug sensitivity, breast cancer

## Abstract

**Introduction:**

CD82, a metastasis suppressor, is known to be palmitoylated, yet the functional significance of this modification in drug response remains unclear. This study investigates the role of CD82 palmitoylation in modulating chemosensitivity in triple-negative breast cancer (TNBC) cells.

**Methods:**

A palmitoylation-deficient CD82 mutant (C5A/C74A/C83A) was generated and expressed in MDA -MB-231 breast cancer cells. Protein palmitoylation was assessed via acyl-biotin exchange assay. Subcellular localization was analyzed by co-immunofluorescence with early endosome (EEA1) and vesicular (VPS4A) markers. Apoptosis was evaluated by measuring levels of apoptotic markers. The cytotoxic effect of the CD82 mutation in combination with gefitinib, doxorubicin, paclitaxel, and camptothecin was quantified using TUNEL and Annexin V/PI assays.

**Results:**

The CD82 palmitoylation-deficient mutant exhibited reduced palmitoylation and increased colocalization with EEA1 and VPS4A. This mutation triggered intrinsic apoptosis, as evidenced by elevated levels of Caspase-3, Cleaved-caspase-3, and Cytochrome C. Strikingly, it synergistically enhanced the cytotoxicity of both gefitinib and doxorubicin, significantly increasing apoptosis in treated cells.

**Discussion:**

Our findings reveal a novel role for CD82 palmitoylation in regulating drug-induced apoptosis. Disruption of palmitoylation potentiates chemotherapy-induced cell death, providing a molecular rationale for targeting CD82 palmitoylation as a combinatorial strategy to overcome therapeutic resistance in TNBC.

## Introduction

Tetraspanins, a conserved family of four-transmembrane proteins, orchestrate critical cellular processes, including migration, protein trafficking, and signal transduction, through dynamic membrane network formation (1). CD82 (KAI1), a prominent tetraspanin, functions as a metastasis suppressor by modulating cell adhesion, Wnt signaling, and senescence, while maintaining muscle homeostasis through satellite cell regulation (2).

Emerging evidence implicates CD82 in TLR9 trafficking and AML chemoresistance, where its overexpression activates the PKCα/β1-integrin pathways to confer drug resistance. Tetraspanin trafficking involves Rab GTPase-mediated sorting through early endosomes, with palmitoylation serving as a critical determinant for membrane retention versus lysosomal degradation via ESCRT complexes (3–8).

Tetraspanins broadly influence drug sensitivity-CD9 knockdown reduces chemoresistance in breast cancer, whereas CD151 deletion sensitizes cells to gefitinib and camptothecin (9, 10). In breast cancer therapeutics, CD63-positive cancer-associated fibroblasts (CAFs) significantly influence tumor cell sensitivity to tamoxifen via paracrine signaling mechanisms (11). CD81 has been implicated in chemotherapy resistance in hematological malignancies, where it mediates drug tolerance via Bruton’s tyrosine kinase (BTK) signaling in both AML and ALL (12). Emerging evidence has identified CD82 as a critical chemoresistance regulator in AML, where treatment-induced upregulation promotes drug resistance via PKCα/β1-integrin pathway activation. CD82-targeting antibodies synergize with cytarabine to enhance therapeutic efficacy through pathway modulation (13, 14).

Preliminary data revealed that palmitoylation-deficient CD82 mutants (C5A/C74A/C83A) increased apoptotic susceptibility in breast cancer cells, although the mechanistic link between CD82 palmitoylation and drug sensitivity remains unexplored.

We hypothesized that these mutations disrupt membrane stability, amplify endosomal–lysosomal trafficking, and selectively activate intrinsic apoptosis, thereby potentiating chemosensitivity.

This study investigates the hypothesis that palmitoylation-deficient CD82 mutants sensitize triple-negative breast cancer cells to chemotherapy by disrupting membrane microdomains and amplifying intrinsic apoptosis, thereby offering a novel combinatorial therapeutic strategy.

## Materials and methods

### Materials

#### Antibodies

• Anti-CD82 Rabbit IgG (Cat No. ab66400, ab108347), anti-CD82 Mouse IgG (Cat No. ab200843, ab59509) were obtained from Abcam, Cambridge, UK. Anti-PERK Rabbit IgG (Cat No. 24390-1-AP, BS3636), Anti-EEA1 C Rabbit Polyclonal IgG (Cat No. 81245-1-RR, BS6299), Anti-VPS4A Rabbit Monoclonal IgG (Cat No. 14272-1-AP, BS80712) were obtained from Bioworld Biotechnology (Wuhan, China).

#### Experimental equipment

• Clean bench (Beijing Medical Equipment Factory), optical microscope (Olympus Corporation of Japan), inverted fluorescence microscope (Leica), upright high-resolution imaging system (Leica), SpectraMax microplate reader (Molecular Devices), Western Blot electrophoresis tank (BIO-RAD), Western Blot transfer electrophoresis instrument (BIO-RAD), −80 °C freezer (Haier, Qingdao), Odyssey fluorescence scanning imaging system (LI-COR), electronic balance (Shenyang Longteng Instrument Factory), manual pipette (Thermo Fisher), microliter injector (Shanghai Gaoge Industry and Trade Co), ultrasonic cell disruptor (USA, Qsonica), benchtop refrigerated incubator shaker (Taicang Huamei Biochemical Instrument Factory), PH meter (OHAUS), and flow cytometer (Agilent).

#### Construction of CD82 palmitoylation mutant

Design and reverse PCR amplification of mutant sites: Using site-directed mutagenesis based on the wild-type CD82 contracted plasmid, we constructed single-point mutations, double-point mutations and triple mutations of KAI1/CD82 plasma proximal membrane cysteine residues (Cys5, Cys74, and Cys83) of palmitoylation to alanine for protein structure stability. Next, primers were designed for the 5` end connection and 3` end in the opposite direction (**Table 1**).

**Table 1:** Sequences of oligonucleotide primers used to construct the site-directed mutations of CD82.

Gene	Primer
Cys5	F: 5’-ATGGGCTCAGCC**GCT**ATCAAAGTCACC-3’ R: 5’-GGTGACTTTGATAGCGGCTGAGCCCAT-3’
Cys74	F: 5’-GGCTTCCTGGGC**GCT**ATCGGCGCCGTC-3’ R: 5’-GACGGCGCCGATAGCGCCCAGGAAGCC-3’
Cys83	F: 5’-AACGAGGTCCGC**GCC**CTGCTGGGGCTG-3’ R: 5’-CAGCCCCAGCAGGGCGCGGACCTCGTT-3’

Primers were designed to mutate CD82 codons C5/74/83 from cysteine to alanine via overlap PCR.

### Cell culture and transfection

The breast cancer cell line (MDA-MB-231) was purchased from the Institute of Biochemistry and Cell Biology of the Chinese Academy of Sciences (Shanghai, China). Cultured cells were grown in dishes (10 cm) or plates (six wells) in DMEM (HyClone, LA, USA) supplemented with 10% FBS (tBD Bioscience, Bristol, UK), antibiotic-100 U/ml penicillin, antibiotic-streptomycin (100) (Beyotime) in humidified air at 37 °C with 5% CO_2_. Plasmids overexpressing CD82, CD82**^C5A/74/83A^** (GenePharm, Shanghai, China) were transfected into breast cancer cell lines (MDA-MB-231) at 40% confluence. After 48 h, the infected cells were harvested.

### Protein extraction and protein immunoblot analysis

Total protein was extracted from cultured cells using RIPA Lysis Buffer (Beyotime, Shanghai, China). Protein concentration was measured using a BCA Protein Assay Kit (Beyotime, Shanghai, China). The protein was immunoblotted with 1:1,000 antibodies against CD82 and other antibodies (Abcam, USA) according to the manufacturer’s instructions. β-actin or GAPDH served as an internal control. The blots were detected using a Bio-Rad Bioimaging system (Bio-Rad, CA, USA). Western blotting was performed as described previously.

### Cell growth assay (CCK8)

Cells were seeded at a density of 5,000 cells per well in 100 μL of suspension in a 96-well plate, with 200 μL of PBS in the peripheral wells. Different concentrations of the drug were added to the plate to establish a concentration gradient, with three to six replicates for each concentration. Three blank wells (without cells) were set up and culture medium containing 10% CCK8 was added. After incubation for 48 h (or 24 h), the culture medium was removed and replaced with medium containing 10% CCK8 and incubated for another 2 h–4 h. The absorbance was measured at 450 nm using a microplate reader.

### TUNEL experiment

The cells were transfected for 48 h, fixed with 4% paraformaldehyde for 30 min at room temperature, washed three times with PBS, and shaken slowly for 5 min each time. A total of 5% Trition X-100 was fixed for 30 min at room temperature, washed three times with PBS; TdT labeling buffer was prepared before use according to the number of samples to be analyzed, 1 μL of TdT enzyme per well, 10 μL of Equilibration buffer, 5 μL of label Mix Green, 34 μL of dd H_2_O per well. Add 5 μL of reaction mixture to each sample and incubate at 37° for 60 min; wash three times with PBS for 5 min each time; add anti-burst fluorescent blocker containing DAPI and observe by fluorescence microscope.

### Acyl-biotinyl exchange assay

After transfecting with CD82 mutant plasmid, culture until cell count reaches ~1 × 10^7^. The cells were washed three times with pre-cooled PBS, scraped, and centrifuged at room temperature to collect them. Lysis buffer (1% Triton×-100 and NEM) was prepared, and the cells were lysed overnight at 4 °C. The next day, the Protein A/G magnetic beads were incubated with the CD82 primary antibody (IgG was used as a control). After incubation, the beads were washed. The lysate was centrifuged, and the supernatant was used as the input group to quantify the protein. The lysate was incubated with the beads for 3 h. Wash the beads again. The HAM solution was prepared and incubated with beads to cleave thioester bonds and generate free thiols. BMCC solution was then prepared and incubated with the beads to convert free thiols to biotin. Finally, the beads were treated with 1 × SDS, boiled to denature the proteins, and the supernatant was collected for WB analysis of palmitoylation levels.

### Annexin V-FITC/PI double staining assay

After inducing apoptosis according to the experimental protocol, the cells were centrifuged at 300×*g* for 5 min, the supernatant was discarded, the cells were washed once with PBS, and the cells were gently resuspended and counted. Take 1–5 × 10^5^ resuspended cells, centrifuge again at 300×*g* for 5 min, discard the supernatant, and resuspend the cells in 500 μL of diluted 1×Annexin V Binding Buffer. Then, 5 μL of Annexin V-FITC and 5 μL of PI (50 μg/mL) were added, mixed gently by vortexing, and incubated at room tempThe cells were washed three times with pre-cooled PBS, serature in the dark for 15 min–20 min. After incubation, the cells were immediately detected using a flow cytometer. If immediate detection is not possible, place the sample on ice, avoid light exposure, and complete the detection within 1 h.

### Immunofluorescence staining

Cells were fixed with 4% paraformaldehyde (PFA) for 15 min and 1% NP-40 for 20 min at 25°C, and then washed three times with PBS. Subsequently, 5% normal goat serum was used for blocking at 37°C for 1 h. The cells were incubated with rabbit anti-EEA1 or anti-VPS4A (1:100; Bioworld) primary antibody (1:100; Bioworld) overnight at 4°C, followed by incubation with fluorophore-conjugated secondary antibodies (1:50; ABclonal) for 1 h. The cell nuclei were stained with DAPI (Beyotime), and the cells were mounted on cover glasses with an antifade mounting medium. Imaging was performed using a Leica Tcssp8 laser scanning confocal microscope (Leica, Wetzlar, Germany) or a Leica DM4B positive fluorescence microscope (Leica).

### Statistical analyses

All the results were repeated several times (≥3) and consistently similar results were collected for statistical analyses. Image J and GraphPad Prism 6 were used to analyze the data. SPSS software was used for two-way ANOVA to analyze the synergy between drugs and mutations. The results are expressed as mean ± standard error of the mean. Note: “*” indicates that compared with the Blank group, P <0.05, “**” indicates that compared with the Blank group (P <0.01), “***” means compared with the Blank group (P <0.001).

## Results

### Mutation of CD82^C5A/C74A/C83A^ reduce palmitoylation and membrane stability

**Figure 1:**
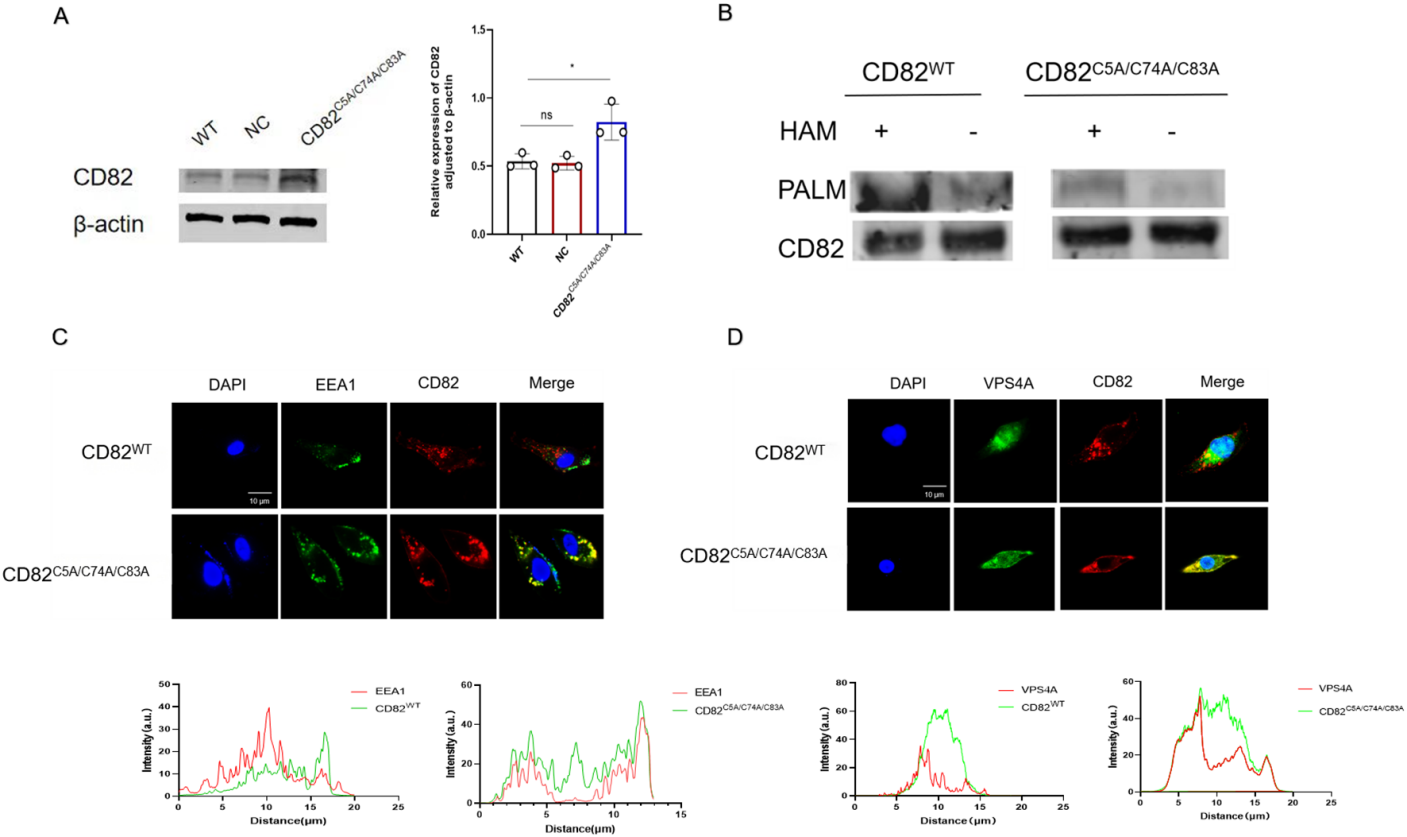
Mutation of CD82 at Cys5, Cys74, and Cys83 reduce palmitoylation levels and membrane stability in breast cancer cells. **(A)** Plasmid transfection induced overexpression of the CD82^C5A/C74A/C83A^ mutant. **(B)** The acyl-biotin switch assay (ABE) was used to assess the palmitoylation levels of CD82 following the CD82^C5A/C74A/C83A^ mutation. **(C, D)** Immunofluorescence assays examined the fluorescent colocalization of CD82 with the early endosome marker EEA1 and the intracellular vesicle marker VPS4A in MDA-MB-231 breast cancer cells after the CD82^C5A/C74A/C83A^ mutation.

The CD82^C5A/C74A/C83A^ overexpression plasmid significantly increased CD82 expression in MDA-MB-231 cells after 48-hour transfection (**Figure 1A**).The ABE assay showed high palmitoylation levels in CD82^WT^ cells but a significant reduction in the CD82^C5A/C74A/C83A^ mutant after transfection of MDA-MB-231 cells (**Figure 1B**). The CD82^C5A/C74A/C83A^ mutation reduces palmitoylation, potentially destabilizing membranes and enhancing recycling via the early endosomal vesicle pathway. Immunofluorescence revealed CD82 co-localization with EEA1 and VPS4A in mutant but not in wild-type cells. This suggests that impaired palmitoylation promotes CD82 trafficking via the recycling pathway, leading to increased apoptosis (**Figures 1C, D**).

### Mutation of CD82^C5A/C74A/C83A^ promotes apoptosis through the mitochondrial pathway

**Figure 2:**
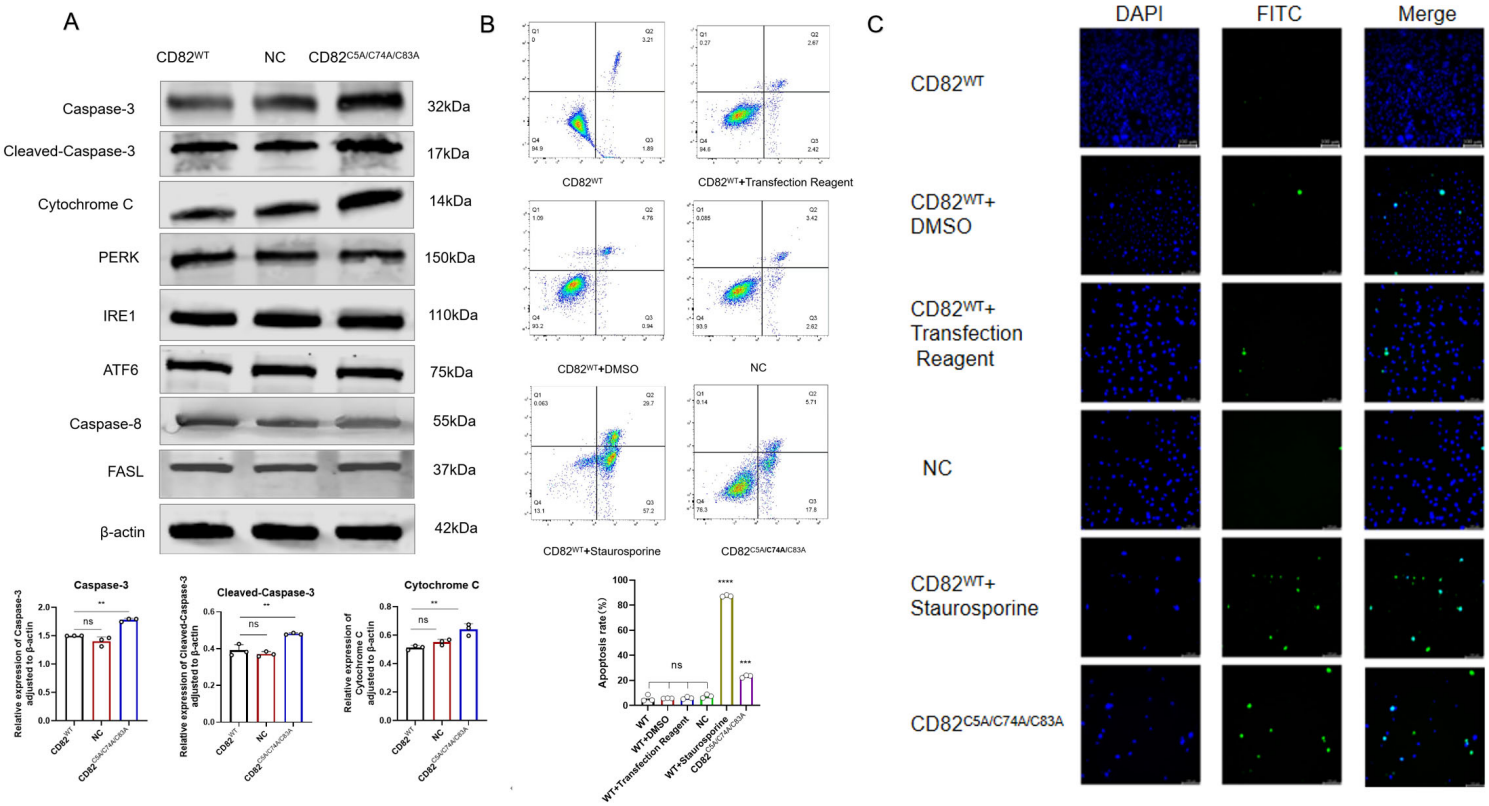
Mutation of CD82 at Cys5, Cys74, and Cys83 exhibit increased apoptosis in breast cancer cells. **(A)** Mitochondrial pathway related apoptotic proteins: Caspase-3, Cleaved-caspase-3, and Cytochrome C were detected. ER pathway related apoptotic proteins: PERK, IRE1, and ATF6 were analyzed. Death receptor pathway related apoptotic proteins: Caspase-8 and FasL were examined. **(B)** An apoptosis assay using AnexinV-FITC/PI double staining was performed. **(C)** The TUNEL assay was also conducted to assess apoptosis. Images were captured at ×100 magnification. Scale bar = 10 μm.

CD82^C5A/C74A/C83A^ and staurosporine (positive control) significantly increased apoptosis in MDA-MB-231 cells compared to controls (DMSO, transfection reagent, empty vector, and CD82^WT^), as shown by Annexin V-FITC/PI (**Figure 2B**) and TUNEL assays (**Figure 2C**). These results suggest that CD82**^C5A/C74A/C83A^** promotes apoptosis in breast cancer cells.

Western blot analysis demonstrated a marked upregulation of Caspase-3, Cleaved-caspase-3, and Cytochrome C in CD82C5A/C74A/C83A mutants, strongly suggesting the activation of mitochondrial-mediated apoptotic pathways. No significant changes were observed in endoplasmic reticulum stress markers (PERK, IRE1, and ATF6) or death receptor pathway components(Caspase-8,FasL) (**Figure 2A**).These findings suggest that CD82^C5A/C74A/C83A^-induced apoptosis is independent of the endoplasmic reticulum or death receptor pathways. Collectively, these results demonstrate that CD82**^C5A/C74A/C83A^** specifically activates the intrinsic mitochondrial apoptosis pathway.

### The combination of the mutation of CD82^C5A/C74A/C83A^ with chemotherapeutic agents (camptothecin, gefitinib, paclitaxel, doxorubicin) was found to enhance apoptosis in breast cancer cells

**Figure 3:**
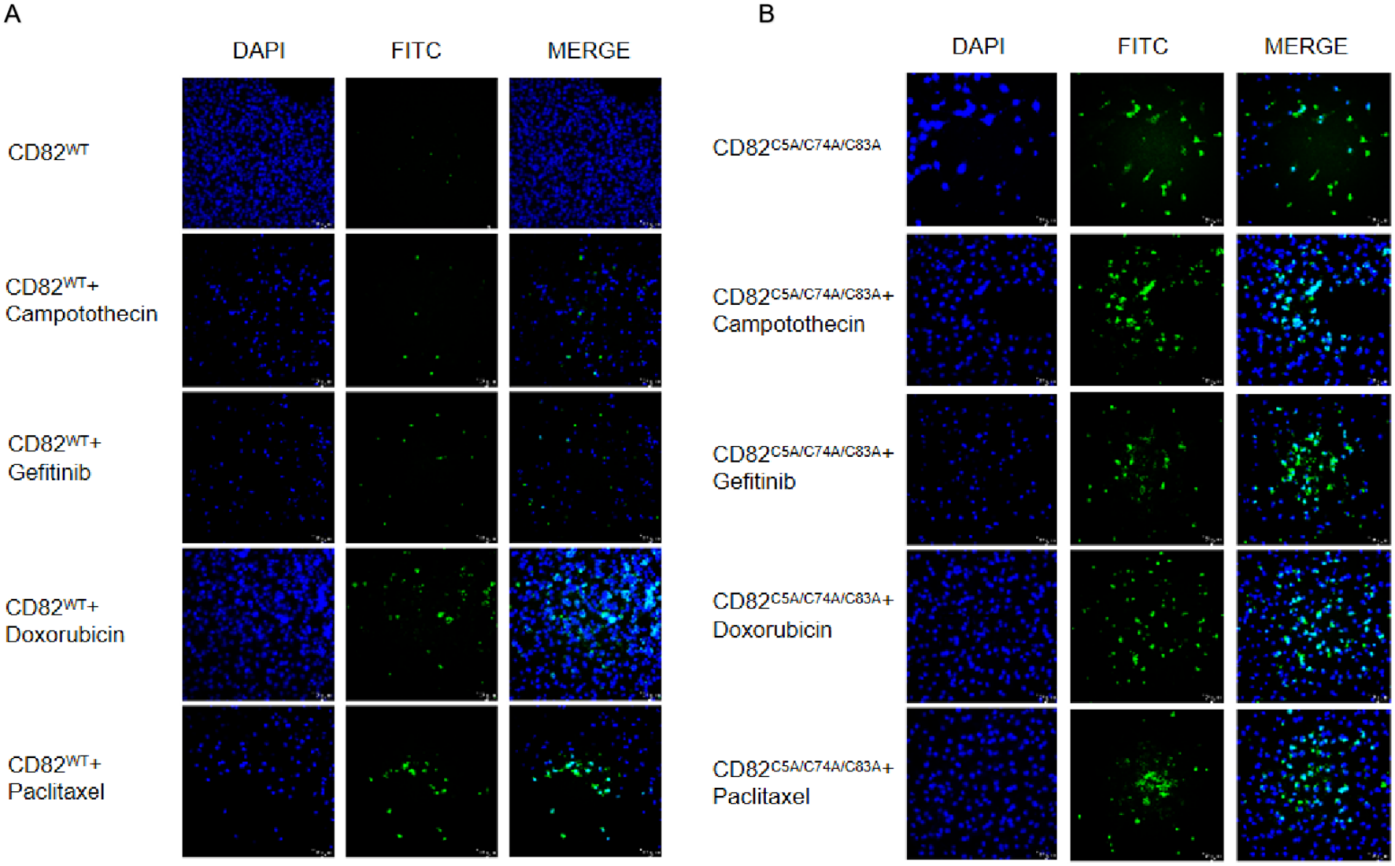
The combination of the mutation of CD82 at Cys5, Cys74, and Cys83 with chemotherapeutic agents (camptothecin, gefitinib, paclitaxel, doxorubicin) was found to enhance apoptosis in breast cancer cells. **(A)** Apoptosis was detected by TUNEL assay in CD82 ^WT^ cells and CD82 ^WT^ cells treated with camptothecin, gefitinib, paclitaxel, or doxorubicin. Scale bar = 10 μm. **(B)** apoptosis was assessed by TUNEL assay in CD82^C5A/C74A/C83A^ mutant cells and mutant cells treated with the same drugs. Scale bar = 10 μm.

TUNEL assay revealed minimal apoptotic activity in CD82^WT^ cells, whereas chemotherapeutic treatment (camptothecin, gefitinib, paclitaxel, and doxorubicin) induced significant apoptosis. Notably, CD82^C5A/C74A/C83A^ cells exhibited substantial baseline apoptosis, which was further enhanced upon drug exposure compared to wild type cells. Strikingly, drug-treated CD82^C5A/C74A/C83A^ cells demonstrated significantly higher apoptotic rates than drug-treated CD82^WT^ cells, suggesting synergistic effects between the CD82 mutation and chemotherapy (**Figures 3A, B**).

### Mutation of CD82^C5A/C74A/C83A^ showed an additive effect with camptothecin and paclitaxel in promoting apoptosis in breast cancer cells

**Figure 4:**
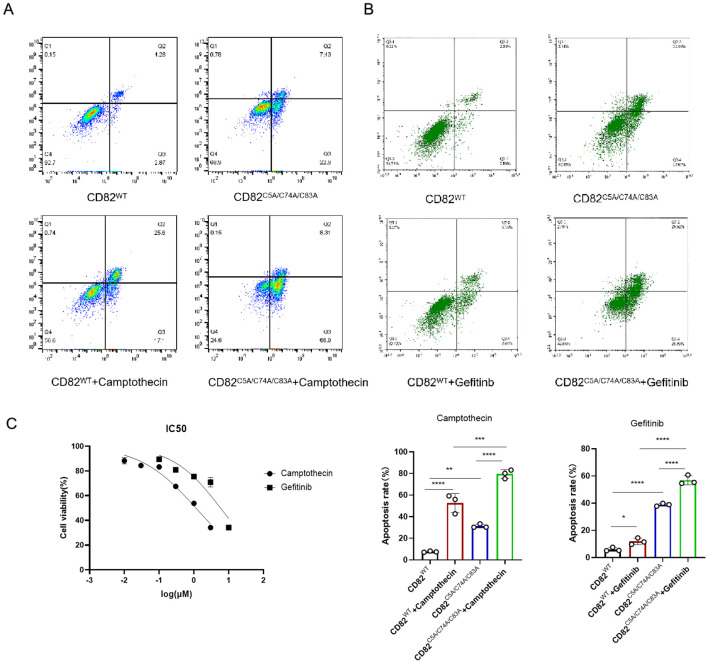
Mutation of CD82 at Cys5, Cys74, and Cys83 showed an additive effect with camptothecin and a synergistic effect with gefitinib in inducing breast cancer cell apoptosis. **(A, B)** Annexin V-FITC/PI double-staining flow cytometry detected apoptosis rates in four groups: CD82 **^WT^**, CD82**^C5A/C74A/C83A^**, CD82^WT^ with 1.5μM Camptothecin, and CD82**^C5A/C74A/C83A^** with 10 μM gefitinib. Statistical analysis covered three biological replicates. *p <0.05, **p <0.01, ***p <0.001, ****p <0.0001 *vs*. the control group (statistically significant differences). **(C)** The CCK8 assay measured the half-inhibitory concentrations of camptothecin and gefitinib in MDA-MB-231 cells.

To study whether CD82^C5A/C74A/C83A^ enhances tumor drug sensitivity, we determined the half inhibitory concentrations (IC50) of camptothecin in MDA-MB-231 cells using the CCK8 assay. (**Figure 4C**). We investigated the impact of the CD82^C5A/C74A/C83A^ mutation in combination with drugs on apoptosis. CD82^C5A/C74A/C83A^ enhanced apoptosis in combination with camptothecin; however, this effect was strictly additive rather than synergistic (**Figure 4A**).

**Figure 5:**
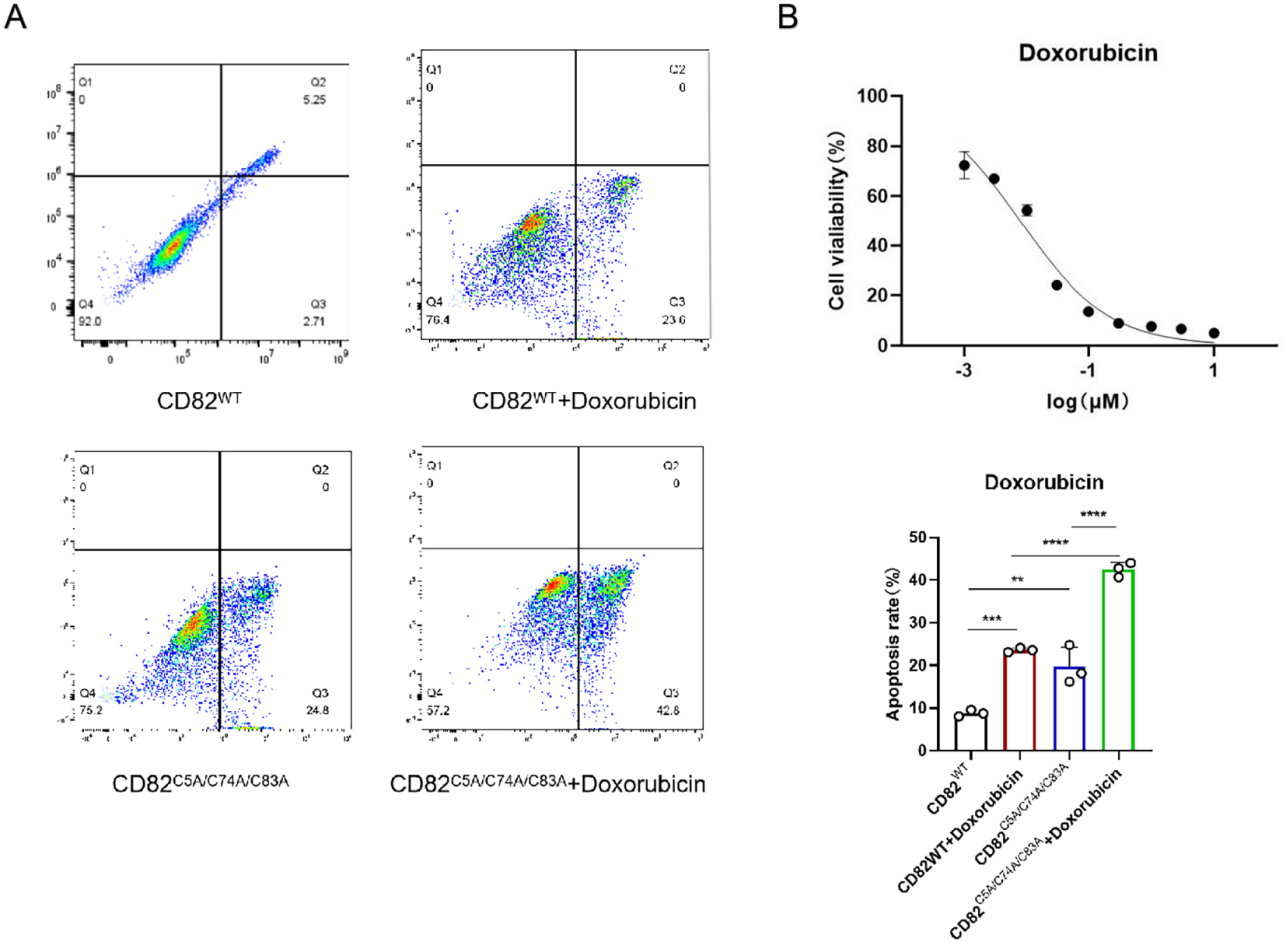
Mutation of CD82 at Cys5, Cys74, and Cys83 exhibits a synergistic effect with doxorubicin in promoting apoptosis in breast cancer cells. **(A)** Annexin V-FITC/PI double - staining flow cytometry was used to assess apoptosis. **(B)** The half-inhibitory concentration of doxorubicin in MDA-MB-231 cells was determined by CCK8 assay. **p < 0.01, ***p < 0.001, ****p < 0.0001.

**Figure 6:**
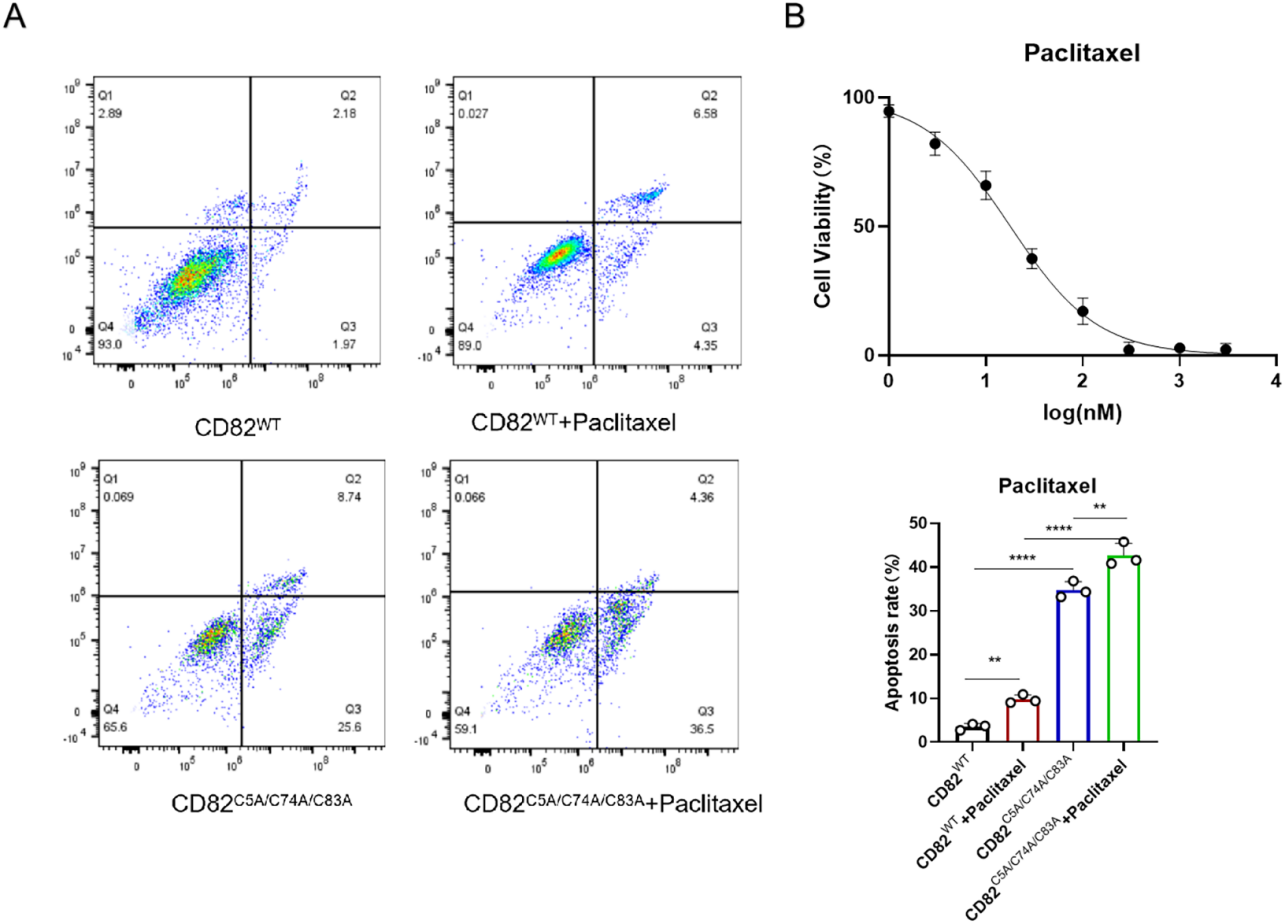
Mutation of CD82 at Cys5, Cys74, and Cys83 exhibits an additive effect with paclitaxel in promoting apoptosis in breast cancer cells. **(A)** Apoptosis rates were assessed by Annexin V-FITC/PI double-staining flow cytometry in four groups: CD82^WT^, CD82^C5A/C74A/C83A^. **(B)** The half-inhibitory concentration of paclitaxel in MDA-MB-231 cells was measured by CCK8 assay.

To explore whether the CD82^C5A/C74A/C83A^ mutation enhances tumor drug sensitivity, we determined the IC50 of paclitaxel in MDA-MB-231 cells using the CCK8 assay (**Figure 6B**). The CD82^C5A/C74A/C83A^ mutation combined with paclitaxel significantly enhanced apoptosis compared to either treatment alone (**Figure 6A**), although their interaction remained additive rather than synergistic. These findings suggest that although the CD82^C5A/C74A/C83A^ mutation potentiates paclitaxel-induced cell death, it does not engage cooperative mechanisms to achieve true synergy.

### Mutation of CD82^C5A/C74A/C83A^ exhibits a synergistic effect with gefitinib and doxorubicin in promoting apoptosis in breast cancer cells

To explore whether the CD82^C5A/C74A/C83A^ mutation enhances tumor drug sensitivity, we used the CCK8 assay to determine the half inhibitory concentration (IC50) of gefitinib and doxorubicin using the CCK8 assay in MDA-MB-231 cells (**Figures 4C**, **5B**).

In gefitinib-treated cells, CD82^C5A/C74A/C83A^ potentiated apoptosis induction beyond additive expectations, revealing a synergistic interaction between the mutation and the tyrosine kinase inhibitor (**Figure 4B**). This indicates that the CD82^C5A/C74A/C83A^ mutation increases the sensitivity of MDA-MB-231 cells to gefitinib.

The CD82**^C5A/C74A/C83A^** mutation synergistically enhanced doxorubicin-induced apoptosis in MDA-MB-231 cells, with the combination treatment demonstrating significantly greater cytotoxicity than either treatment alone (**Figure 5A**).

These synergistic interactions suggest that the mutation may sensitize triple-negative breast cancer cells to anthracycline-based chemotherapy, potentially offering a therapeutic strategy for treatment-resistant cases.

## Discussion

CD82 (KAI1) is a metastasis suppressor that inhibits tumor progression through multiple mechanisms, including the attenuation of cell motility, maintenance of cellular polarity, and induction of apoptosis in response to extracellular stimuli, while also regulating protein trafficking and membrane dynamics (15, 16). As a tetraspanin, CD82 critically maintains membrane stability and cell migration, with its palmitoylation sites being essential for these functions, as their mutation disrupts membrane homeostasis. Following internalization, CD82 undergoes endosomal sorting via recycling pathways, a process essential for cellular homeostasis, and its dysregulation contributes to disease pathogenesis (17).

S-palmitoylation, a reversible post-translational modification mediated by ZDHHC enzymes and APTs, regulates diverse cellular processes including signal transduction and membrane trafficking, with dysregulation implicated in cancer pathogenesis through modulation of oncoproteins and tumor suppressors (18–20). The dynamic regulation of protein palmitoylation in cancer cells through altered expression of ZDHHCs/APTs represents a promising therapeutic target for modulating drug sensitivity and overcoming chemoresistance in multiple signaling pathways (21).

Our ABE assays confirmed that the C5A/C74A/C83A mutations effectively reduced CD82 palmitoylation, aligning with prior reports that these residues critically maintain tetraspanin web integrity and providing mechanistic insight into the apoptotic phenotype. The enhanced drug sensitivity resembles AML models, where CD82 targeting improved cytarabine efficacy, but differs mechanistically by exploiting intrinsic apoptosis rather than PKCα/β1-integrin modulation, highlighting tissue-specific pathway engagement. These observations may arise via several non-exclusive mechanisms, including palmitoylation loss disrupting CD82-Rab11a/FIP2 complexes and their survival signal recycling function, mutant accumulation in endosomes overloading vesicular quality control, or altered membrane fluidity indirectly activating BAX/BAK (22, 23).

While tetraspanins are known to modulate drug resistance, this study is the first status to directly link to chemosensitivity, demonstrating synergistic effects with gefitinib and doxorubicin. The mitochondrial apoptosis preference contrasts with CD82’s established roles in membrane microdomain organization, suggesting that palmitoylation loss may trigger distinct downstream effects. Wild-type CD82 supports metastasis suppression through adhesion modulation, whereas the mutants induce cytotoxicity via organelle stress pathways. Emerging evidence reveals that protein palmitoylation critically modulates drug sensitivity, with PD-L1 hyperpalmitoylation driving cisplatin resistance in bladder cancer by stabilizing immune checkpoint signaling, while ZDHHC22-mediated palmitoylation overcomes endocrine resistance in ER-negative breast cancer via mTOR destabilization and AKT pathway suppression (24), establishing palmitoylation as both a predictive biomarker and therapeutic target for treatment-resistant malignancies.

Clinically, these findings suggest that the CD82 palmitoylation status could be used to stratify patients for combination therapies, particularly in TNBC, where mitochondrial apoptosis pathways remain targetable and current treatments lack precision. Limitations include uncharacterized effects on other tetraspanin network members that may contribute to the phenotype and the need for *in vivo* validation of the chemosensitization effect. Future studies should map the precise molecular cascade linking palmitoylation-deficient CD82 to mitochondrial outer membrane permeabilization and explore whether this mechanism extends to other therapy-resistant cancers.

**Figure 7:**
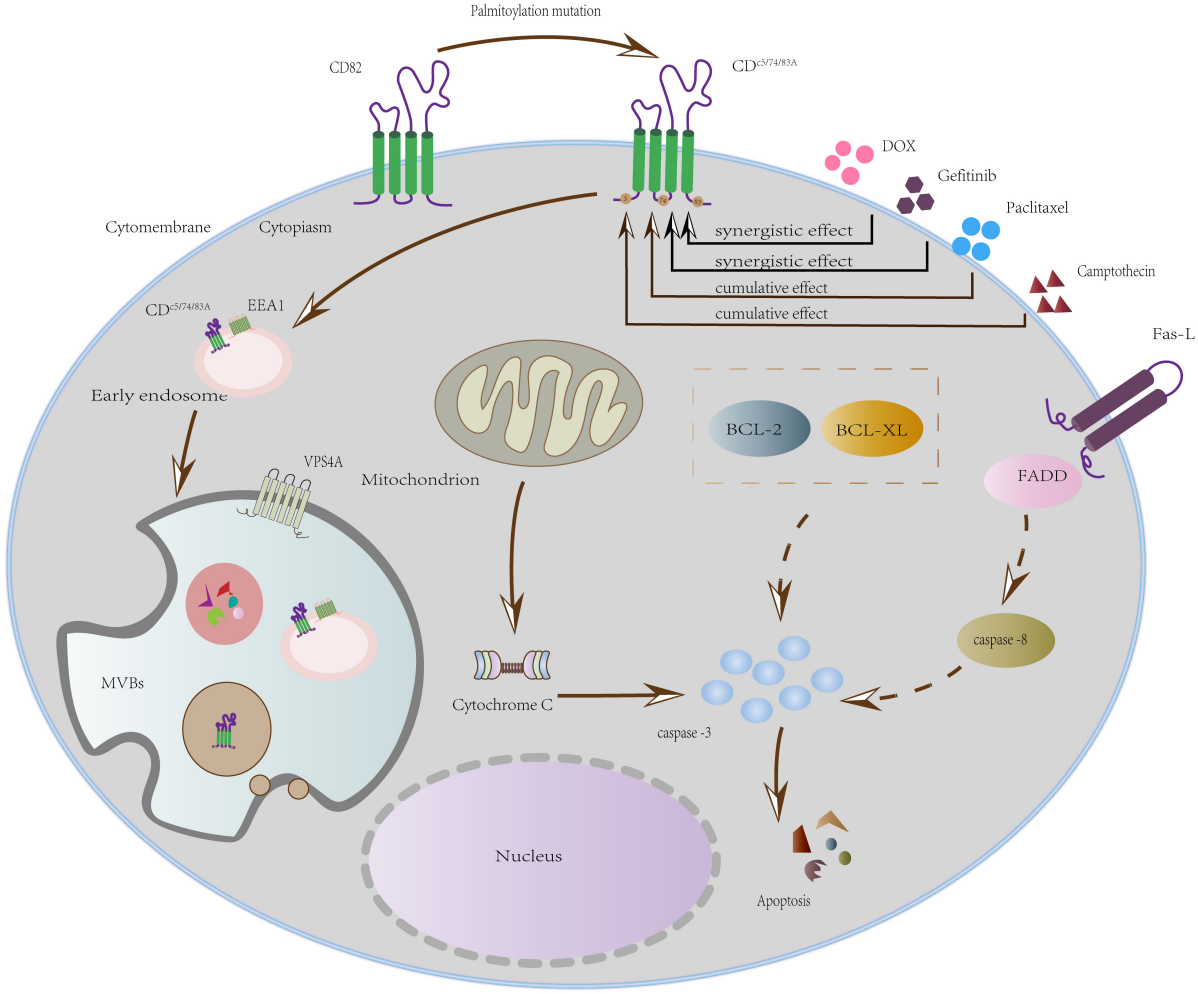
Mechanism of synergistic apoptosis induction by DOX/gefitinib and a CD82 mutant.

The schematic (**Figure 7**) delineates how DOX and gefitinib induce mitochondrial apoptosis by mediating MOMP, cytochrome c release, and caspase activation. We propose that the internalization of CD82 (via triple-palmitoylation-site mutation) functions as a cellular sensitization mechanism for this intrinsic pathway, thereby explaining the synergistic effect of this mutant with doxorubicin (DOX) and gefitinib.

While this study establishes a mechanistic rationale for tetraspanin-targeted combination therapies, the precise metabolic rewiring and upstream regulators governing palmitoylation-deficient tetraspanin-induced apoptosis remain to be fully characterized. Future studies should delineate how tetraspanin palmitoylation status integrates with oncogenic signaling networks to advance both fundamental understanding and therapeutic translation.

## Data Availability

The datasets presented in this study can be found in online repositories. The names of the repository/repositories and accession number(s) can be found in the article/**Supplementary Material**.
