# Supplementary figures and images for: Mechanistic role of CD82 palmitoylation in augmenting antitumor drug sensitivity via apoptosis regulation
Source: Front Oncol. 2025 Dec 3;15:1699420. doi: 10.3389/fonc.2025.1699420 (PMC12708250; doi:10.3389/fonc.2025.1699420)

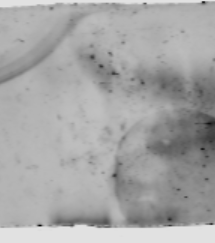

Supplement: Supplementary file 1 [file DataSheet1.zip › WB raw data/ABE-7.tif]

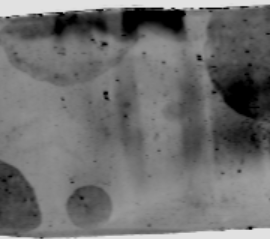

Supplement: Supplementary file 1 [file DataSheet1.zip › WB raw data/ABE-WT.tif]

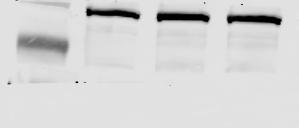

Supplement: Supplementary file 1 [file DataSheet1.zip › WB raw data/ATF6.jpg]

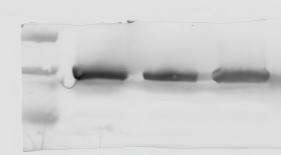

Supplement: Supplementary file 1 [file DataSheet1.zip › WB raw data/Caspase-8.jpg]

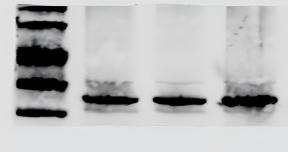

Supplement: Supplementary file 1 [file DataSheet1.zip › WB raw data/Cleaved-Caspase-3.jpg]

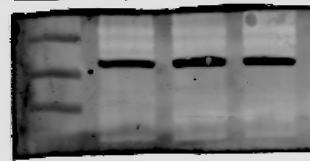

Supplement: Supplementary file 1 [file DataSheet1.zip › WB raw data/Fasl.jpg]

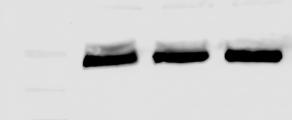

Supplement: Supplementary file 1 [file DataSheet1.zip › WB raw data/IRE1.jpg]

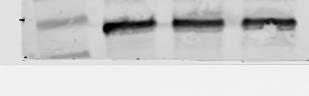

Supplement: Supplementary file 1 [file DataSheet1.zip › WB raw data/PERK.jpg]

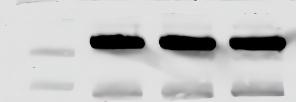

Supplement: Supplementary file 1 [file DataSheet1.zip › WB raw data/β-actin.jpg]
